# Evolution of transplant oncology indications: a single-institution experience over 40 years

**DOI:** 10.1007/s13304-024-01827-1

**Published:** 2024-04-08

**Authors:** Quirino Lai, Simona Parisse, Stefano Ginanni Corradini, Flaminia Ferri, Konstantina Kolovou, Pasquale Campagna, Fabio Melandro, Gianluca Mennini, Manuela Merli, Massimo Rossi

**Affiliations:** 1 General Surgery and Organ Transplantation Unit, Department of General and Specialty Surgery, Sapienza University of Rome, AOU Policlinico Umberto I of Rome, Rome, Italy; 2 Department of Translational and Precision Medicine, Sapienza University of Rome, AOU Policlinico Umberto I of Rome, Rome, Italy

**Keywords:** Colorectal liver metastases, Hepatocellular carcinoma, Peri-hilar cholangiocellular carcinoma, Intra-hepatic cholangiocellular carcinoma, Hemangioendothelioma

## Abstract

Liver transplantation (LT) for uncommon tumoral indications has changed across the decades, with impaired results reported in the first historical series mainly for non-tumoral-related causes. Recently, renewed interest in liver transplant oncology has been reported. The study aims to analyze a mono-center experience exploring the evolution and the impact on patient survival of LT in uncommon tumoral indications. A retrospective analysis of 851 LT performed during 1982–2023 was investigated. 33/851 (3.9%) uncommon tumoral indications were reported: hepatocellular carcinoma (HCC) on non-cirrhotic liver (*n* = 14), peri-hilar (phCCA) (*n* = 8) and intrahepatic cholangiocarcinoma (i-CCA) (*n* = 3), metastatic disease (*n* = 4), hepatic hemangioendothelioma (*n* = 2), and benign tumor (*n* = 2). Uncommon tumoral indications were mainly transplanted during the period 1982–1989, with a complete disappearance after the year 2000 and a slight rise in the last years. Poor outcomes were reported: 5-year survival rates were 28.6%, 25.0%, 0%, and 0% in the case of HCC on non-cirrhotic liver, phCCA, i-CCA, and metastases, respectively. However, the cause of patient death was often related to non-tumoral conditions. LT for uncommon oncological diseases has increased worldwide in recent decades. Historical series report poor survival outcomes despite more recent data showing promising results. Hence, the decision to transplant these patients should be under the risk and overall benefit of the patient. The results of the ongoing protocol studies are expected to confirm the validity of the unconventional tumor indications.

## Introduction

Liver transplantation (LT) represents the best therapy for the cure of a large population of selected patients with end-stage liver diseases and tumors [1]. In transplant oncology, several tumoral diseases are considered eligible for LT [2]. Among them, hepatocellular carcinoma (HCC) developed on cirrhosis represents the most common tumoral indication [3]. However, other less commonly reported tumors should represent an indication for LT, like HCC on normal liver [4], peri-hilar (ph-CCA) or intrahepatic cholangiocarcinoma (i-CCA) [5, 6], metastases [7, 8], and vascular tumors [9]. The excellent results reported in the case of HCC on cirrhosis are well known, with post-LT 5-year overall survival (OS) rates exceeding 70–75% in several series [3, 10]. Conversely, the other tumors showed inferior results [4–9].

The indication for LT in the presence of less common tumors has radically changed across the decades, with impaired results reported mainly in the first historical series, more for management- and technical-related aspects than for high recurrence rates [11].

A recent revision of the monocentric LT experience of the Sapienza University of Rome has been made, focusing attention on the long survivors and the significant evolution observed in the last four decades in the setting of LT [12]. Similarly, this study aims to analyze this center experience by exploring the evolution and impact of patient survival of the uncommon tumoral indications for LT.

## Methods

### Study design

The present study is a retrospective monocentric research based on a prospectively maintained database of patients transplanted in the Azienda Ospedaliero-Universitaria Policlinico Umberto I of Rome, Sapienza University of Rome, Italy. This study followed the Strengthening the Reporting of Observational Studies in Epidemiology (STROBE) reporting guidelines. The institutional review board of Azienda Ospedaliero-Universitaria Policlinico Umberto I approved the study.

### Setting

Participants included the patients undergoing LT in the General Surgery and Organ Transplantation Unit of the Azienda Ospedaliero-Universitaria Policlinico Umberto I of Rome, Sapienza University of Rome, Italy.

### Population

Eight hundred and eleven patients consecutively received 851 LT from May 1982 to September 2023. All the patients transplanted during this period were considered for the study. Therefore, no exclusion criteria were applied.

### Variables and data collection

Data collected in the study included:

- Recipient characteristics = age, sex, period of transplant (1982–1989, 1990–1999, 2000–2009, 2010–2019, 2020–2023), blood group, Caucasian ethnicity, HCC, HCV, HBV, alcohol, non-alcoholic steatohepatitis (NASH) or cryptogenic, biliary cirrhosis, acute liver failure, other liver diseases, cause of death.
- Donor characteristics = age, sex, blood group.
- Transplantation characteristics = split liver, living donation, multiorgan transplantation, urgency transplantation, use of bypass for caval reconstruction, total ischemia time.

Patient death was defined as any transplant-related or unrelated event of death observed at any time from LT. Patient death time was calculated as the time from LT to the death event during the follow-up. Patients alive at the last follow-up were censored. The later follow-up date was September 30, 2023.

In patients with HCC on non-cirrhotic liver, liver parenchyma was analyzed for grading of steatosis according to Brunt et al. [13], for fibrotic state according to Metavir score [14], and for inflammatory activity according to the Histological Activity Index (HAI) proposed by Knodell and Ishak [15].

As for the evolution of immunosuppression regimens during the decades, a more detailed definition of these aspects has been reported in a previous study [12].

### Statistical analysis

Baseline characteristics of each data set were presented as medians and first–third quartile (Q1–Q3) for continuous variables and as numbers and percentages for discrete variables. Comparisons between groups were made using Fisher’s exact test or the chi-square test for categorical variables, as appropriate. Mann–Whitney was used for continuous variables. No missing data relative to study variables were observed; therefore, no data interpolation was required.

Survival curves were performed using the Kaplan–Meier method. A log-rank test was used to compare the survival results.

A *P* value < 0.05 was considered statistically significant. Statistical analyses were conducted using SPSS 27.0 (SPSS Inc., Chicago, IL, USA).

## Results

**Fig. 1:**
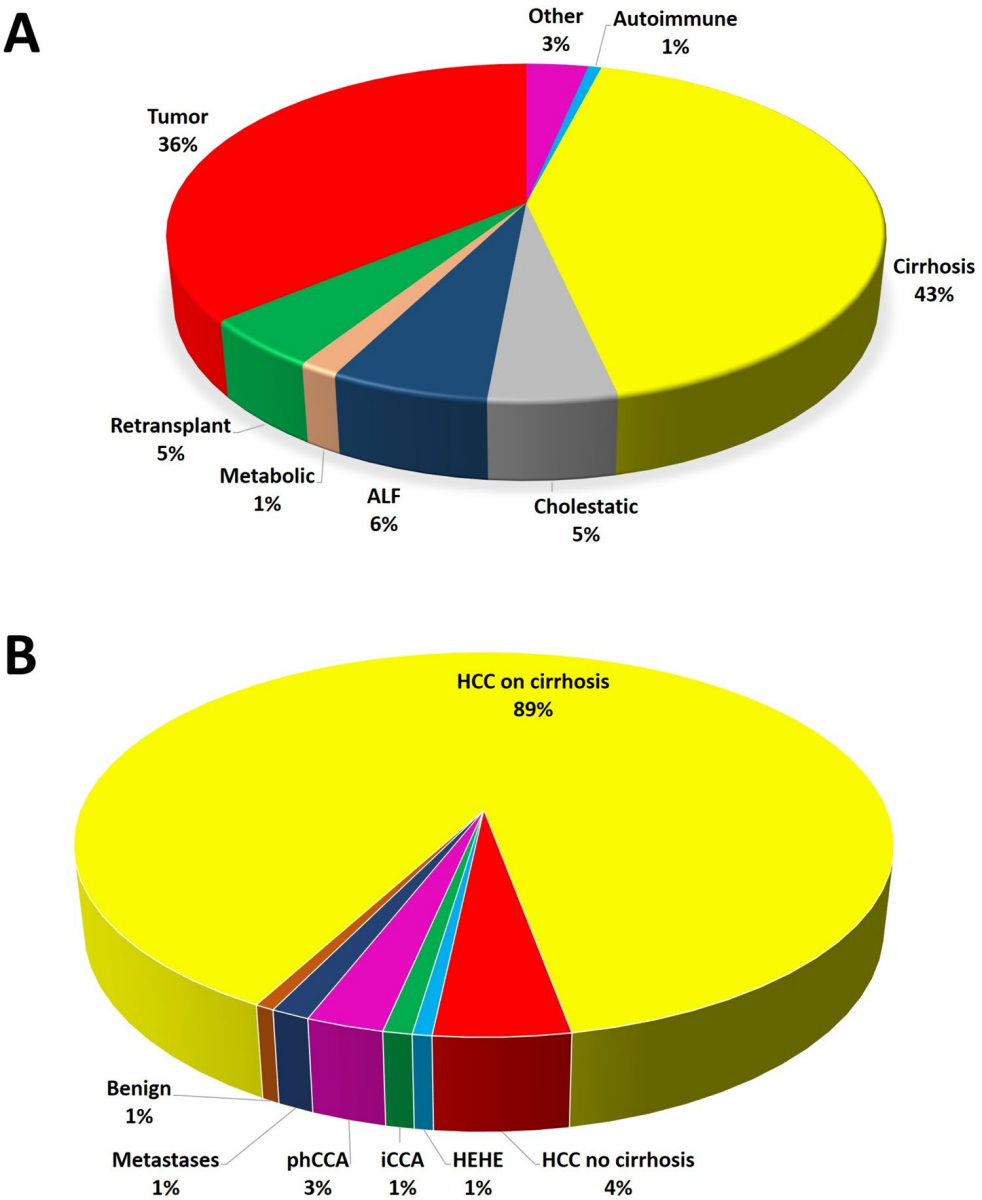
**A** Different main indications for LT reported in the present cohort; **B** different tumor-related indications observed in the present cohort

The median follow-up period for the entire cohort (*N* of patients = 811, *N* of LT = 851) was 5.3 years (Q1–Q3 = 0.5–12.6). After having categorized the entire cohort in different classes according to the main cause of transplantation, the following categories were identified: autoimmune cirrhosis = 6/851 (0.7%), viral- or alcohol- or NASH-related cirrhosis = 362 (42.5%), cholestatic diseases = 42 (4.9%), acute liver failure = 52 (6.1%), metabolic disorder = 13 (1.5%), re-transplantation = 41 (4.8), other disease = 28 (3.3), and tumor = 307 (36.1) (Fig. 1A).

After looking more in detail at the tumor-related causes, HCC on cirrhosis was the main indication (*n* = 274/307, 89.3%), followed by HCC developed on a non-cirrhotic liver (14, 4.6%), phCCA (*n* = 8, 2.6%), metastatic disease (*n* = 4, 1.3%), iCCA (*n* = 3, 1.0%), hepatic hemangioendothelioma (HEHE) (*n* = 2, 0.7%), and benign tumor (*n* = 2, 0.7%) (Fig. 1B).

**Fig. 2:**
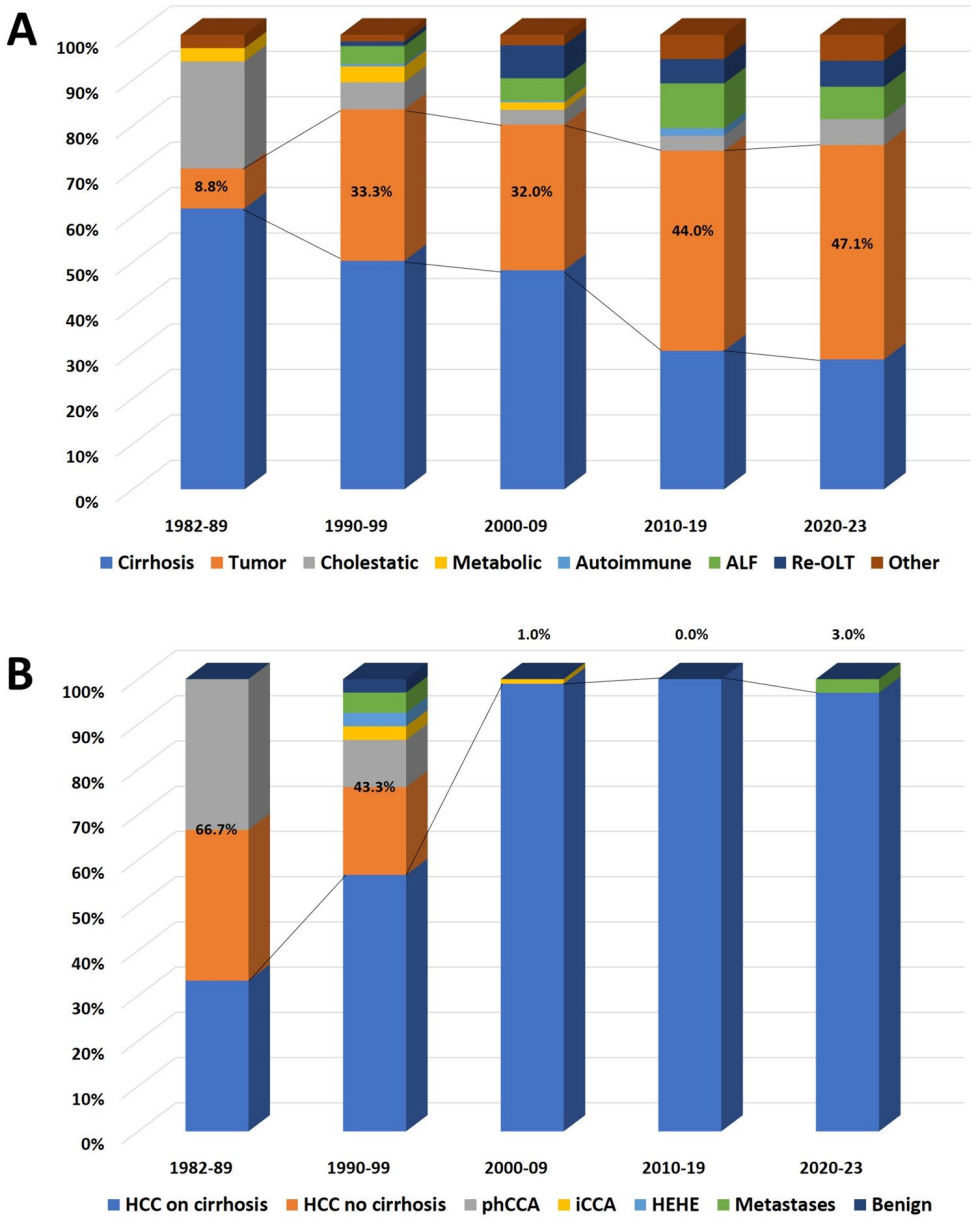
**A** Evolution of the different main indications in the different temporal periods in the present cohort; **B** evolution of the different tumor-related indications in the different temporal periods in the present cohort

Observing the evolution of LT indication across the different decades, it is interesting to observe that the tumoral indication reported a progressive increase in the percentage of transplanted patients, passing from 8.8 to 47.1% of indications in the periods 1982–1989 and 2020–23, respectively (Fig. 2A). As expected, the uncommon tumoral indications were more common in 1982–1989, showing a progressive decline and a complete disappearance after 2000. In detail, the uncommon tumoral indications passed from 66.7% and 43.3% during 1982–1989 and 1990–1999 to 1.0% and zero during 2000–2009 and 2010–2019, respectively. During the last years, this percentage has slightly risen to 3.0% (Fig. 2B).

### Uncommon tumoral indications for transplantation vs. HCC on cirrhosis

The characteristics of the uncommon tumoral indications are reported in Table 1, also reporting a comparison with the group of patients receiving a LT for HCC on cirrhosis.

**Table 1:** Recipient-, donor-, and transplant-related characteristics of patients receiving a LT for HCC on cirrhosis vs. uncommon tumoral disease

Variables	HCC on cirrhosis (*n* = 274)	Other tumors (*n* = 33)	*P*
	Median (Q1–Q3) or *n* (%)		
Recipient			
Age, years	59 (52–63)	44 (40–55)	< 0.0001
Male sex	242 (88.3)	16 (48.5)	< 0.0001
Period of transplantation			
1982–89	1 (0.4)	2 (6.1)	
1990–99	38 (13.9)	29 (87.9)	< 0.0001
2000–09	96 (35.0)	1 (3.0)	
2010–19	107 (39.1)	0 (–)	
2020–23	32 (11.7)	1 (3.0)	
Group			
0	135 (49.3)	14 (42.4)	
A	102 (37.2)	14 (42.4)	0.91
B	22 (8.0)	3 (9.1)	
AB	15 (5.5)	2 (6.1)	
Caucasian ethnicity	265 (96.7)	32 (97.0)	1
Underlying liver disease			
HCV	139 (50.7)	1 (3.0)	< 0.0001
HBV	58 (21.2)	1 (3.0)	0.009
Alcohol	85 (31.0)	0 (–)	< 0.0001
NASH/cryptogenic	30 (10.9)	0 (–)	0.06
Acute liver failure	1 (0.4)	1 (3.0)	0.2
No underlying liver disease	0 (–)	31 (93.9)	< 0.0001
Donor			
Age, years	50 (34–64)	27 (18–43)	< 0.0001
Male sex	139 (50.7)	19 (57.6)	0.47
Group			
0	142 (51.8)	19 (57.6)	
A	101 (36.9)	11 (33.3)	0.7
B	22 (8.0)	3 (9.1)	
AB	9 (3.3)	0 (–)	
Transplantation			
Split liver	2 (0.7)	0 (–)	1
Living donation	6 (2.2)	0 (–)	1
Multivisceral	2 (0.7)	2 (6.1)	0.001
Cluster	0 (–)	1 (3.0)	
Status UNOS 1-2A	4 (1.5)	4 (12.1)	0.006
Bypass and caval replacement	105 (38.3)	32 (97.0)	
Piggy-back	17 (6.2)	1 (3.0)	< 0.0001
Piggy-back LL	152 (55.5)	0 (–)	
Total ischemia time, min	450 (369–520)	660 (540–780)	< 0.0001

*HCC* hepatocellular carcinoma, *Q1–Q3* first-third quartile, *HCV* hepatitis C virus, *HBV* hepatitis B virus, *NASH* non-alcoholic steato-hepatitis, *UNOS* United Network for Organ Sharing, *LL* latero-lateral

As expected, relevant differences were observed between the two groups. In general, patients with uncommon tumoral indications were principally transplanted during the last two decades of the last century (94.0 vs. 14.3%; *P* < 0.0001), being younger (median: 44 vs. 59 years; *P* < 0.0001) and more commonly females (51.5 vs. 11.7%; *P* < 0.0001) respect to patients transplanted for HCC on cirrhosis.

The uncommon tumoral indication typically raised without a contemporaneous underlying liver disease (93.9% vs. no cases; *P* < 0.0001). As for the donor characteristics, grafts coming from younger donors were used in patients transplanted for uncommon tumoral indications (Median: 27 vs. 50 years; *P* < 0.0001). As for the characteristics of the transplantation procedure, patients with uncommon tumoral indications received a total caval replacement in the great majority of the cases (97.0 vs. 38.3%; *P* < 0.0001), while piggy-back techniques were more commonly used in HCC on cirrhosis cases. Multivisceral or cluster procedures were commonly observed in patients with uncommon tumors (9.1 vs. 0.7%; *P* = 0.001). The surgical procedure’s increased complexity impacted the longer total ischemia time (median: 660 vs. 450 min; *P* < 0.0001).

### Specific features of the uncommon tumoral indications

The characteristics of the patients with HCC developed on a non-cirrhotic liver are reported in Table 2. In detail, patients with this type of tumor typically reported more advanced tumors with respect to HCCs on cirrhosis, with a high percentage of macrovascular and microvascular invasion (42.9 and 64.3% of the cases, respectively), poor grading (71.4%), and morphological features of the tumor exceeding the conventional transplantability criteria (100.0% exceeding the Milan Criteria and 85.7% exceeding the University of California San Francisco Criteria). As expected, the percentage of recurrence was high (57.1% of the cases).

**Table 2:** Characteristics of the patients with HCC on non-cirrhotic liver

Variables	HCC on non-cirrhotic liver (*n* = 14) Median (Q1–Q3) or *n* (%)				
Number of lesions	2 (1–3)				
Number of lesions > 3	2 (14.3)				
Diameter of major lesion (cm)	9.5 (6.1–12.3)				
Sum of dimensions (cm)	11.6 (8.5–14.2)				
Sum of dimensions > 5 cm	12 (85.7)				
Sum of dimensions > 8 cm	10 (71.4)				
Multifocality	7 (50.0)				
Bilobar involvement	5 (35.7)				
Macrovascular invasion	6 (42.9)				
Microvascular invasion	9 (64.3)				
Poor grading	10 (71.4)				
Exceeding MC	14 (100.0)				
Exceeding UCSF	12 (85.7)				
Post-LT HCC recurrence	8 (57.1)				

% of steatosis		Metavir fibrosis score		HAI	
Category	*N* (%)	Category	*N* (%)	Grade	*N* (%)
Absence	4 (28.6)	Absence	8 (57.1)	0	4 (28.6)
1: < 33%	2 (14.3)	1: Mild	1 (7.1)	1	1 (7.1)
2: 33–66%	4 (28.6)	2: Moderate	4 (28.6)	2	2 (14.3)
3: > 66%	4 (28.6)	3: Marked	1 (7.1)	3	2 (14.3)
		4: Cirrhosis	0 (–)	4	3 (21.4)
				5	2 (14.3)

Liver parenchyma was analyzed for grading of steatosis according to Brunt et al., for fibrotic state according to Metavir score, and for inflammatory activity according to HAI proposed by Knodell and Ishak

*HCC* hepatocellular carcinoma, *Q1–Q3* first-third quartile, *n* number, *MC* Milan Criteria, *UCSF* University of California San Francisco, *LT* liver transplantation, *HAI* Histological Activity Index

Interestingly, when the non-cirrhotic liver parenchyma was explored, signs of moderate-to-advanced steatosis (*n* = 8, 57.1%), mild-to-marked fibrosis (*n* = 6, 42.9%), and inflammation activity (*n* = 10, 71.4%) were reported, showing that the non-cirrhotic liver often was connected with signs of steatohepatitis.

A total of 13/14 (92.9%) patients died after LT. In detail, HCC recurrence was the leading cause of patient death in 8/14 (57.1%) patients. The remaining five deaths were caused by post-transplant infection (*n* = 2), intraoperative complication (*n* = 1), cardiac event (*n* = 1), and chronic rejection (*n* = 1).

In Table 3, the characteristics of the other uncommon tumoral indications were reported.

**Table 3:** Characteristics of the other uncommon tumor indications

*N* center	Year LT	Age	Sex	Multiorgan technique	Pathological assessment	Follow-up months	Death	Cause death
phCCA								
#33	1989	57	F	–	T3N1Mx G3	0	Yes	Cardiac infarction
#50	1991	28	M	Multivisceral	Hepatic hilum, duodenum, pancreas and mesocolon infiltrated	1	Yes	Bacterial infection
#53	1991	47	F	Cluster	Hepatic hilum, stomach, and duodenum infiltrated	3	Yes	Bacterial infection pancreatitis
#68	1992	36	F	–	T2aN0Mx G2	14	Yes	Tumor recurrence
#73	1992	49	M	–	T2aN0Mx G1	153	Yes	Chronic rejection
#130	1995	60	M	–	T3N1Mx G3	0	Yes	Cardiac infarction
#161	1996	42	F	–	T2aN0Mx G1	169	No	–
#168	1997	52	F	–	T3N1Mx G3	0	Yes	Bacterial infection
iCCA								
#86	1993	52	F	–	T2N0Mx G3	0	Yes	Bacterial infection
#131	1995	42	M	–	T1N0Mx G2	29	Yes	Chronic rejection
#404	2004	57	M	–	T2N0Mx G3	1	Yes	PDF
Metastases								
#51	1991	37	F	Multivisceral	Left colon with massive CRLM and IVC infiltration	4	Yes	Bacterial infection acute rejection
#143	1996	55	M	–	Right renal tumor with unresectable liver metastases G1	18	Yes	Recurrence
#181	1997	40	F	–	Unresectable CRLM	1	Yes	GI hemorrhage
#821	2022	54	F	–	Unresectable CRLM (MELODIC protocol)	18	No	–
HEHE								
#97	1994	44	M	–	Microvascular and glissonian invasion, high mitotic activity	19	Yes	Recurrence
#231	1999	26	F	–	No microvascular and glissonian invasion, low mitotic activity	286	No	–
Benign								
#64	1992	42	F	–	Adenomatosis	362	No	–
#169	1997	39	F	–	Giant hemangioma	296	No	–

*N* number, *LT* liver transplantation, *phCCA* peri-hilar cholangiocellular carcinoma, *F* female, *M* male, *iCCA* intrahepatic cholangiocellular carcinoma, *PDF* primary dysfunction, *CRLM* colorectal liver metastases, *IVC* inferior vena cava, *GI* gastrointestinal, *HEHE* hepatic hemangioendothelioma

Among the eight cases of phCCA transplanted, two cases of long survivors were reported, with survivals exceeding 12 and 14 years from LT. Interestingly, only one recurrence was observed, while all the other cases died within the first 3 months after transplant due to infections or cardiac events.

The three cases of iCCA all ended in patient death. However, no case of death due to recurrence was reported: the only patient surviving more than 2 years from LT died due to graft loss for chronic rejection.

As for the metastases, three cases were transplanted before 2000, all showing adverse outcomes. Two colorectal liver metastases (CRLM) patients died within the first 4 months after LT due to bacterial infection and gastrointestinal hemorrhage. One case transplanted due to renal metastases survived 18 months, dying of tumor recurrence. The only case transplanted for CRLM in the present century has been enrolled in the MELODIC protocol: the patient is alive more than 1 year after transplantation.

A long survivor with HEHE (i.e., approximately 24 years) was reported. Another HEHE case with more aggressive tumoral behavior (microvascular and Glissonian invasion) recurred 19 months after LT.

Lastly, the two cases transplanted for benign tumoral disease (i.e., adenomatosis and giant hemangioma) are alive and long survivors after transplantation (i.e., more than 30 and 24 years, respectively).

### Survival rates

**Fig. 3:**
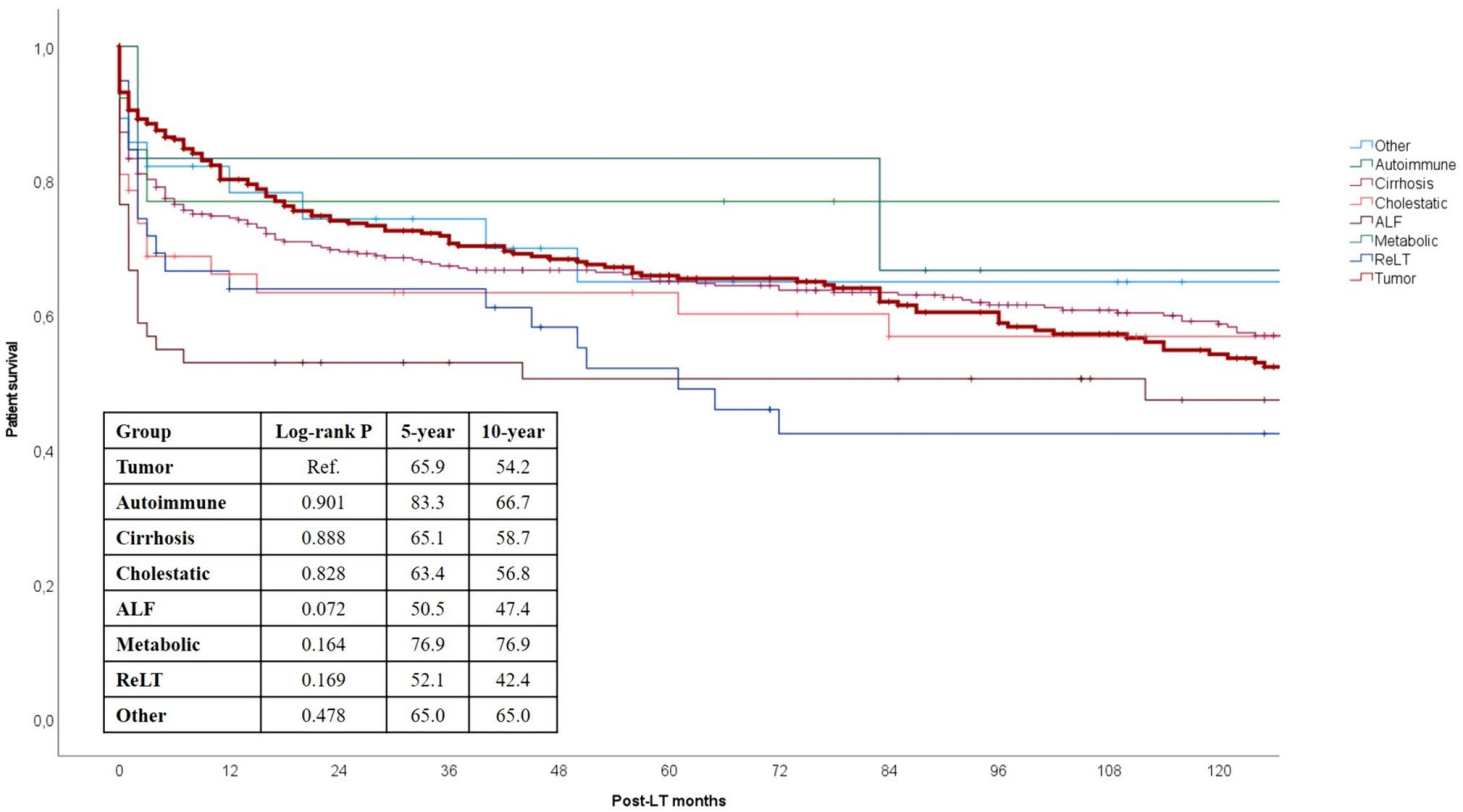
Long-term survival rates stratified according to the different main indications for LT

In Fig. 3, the long-term survival rates of different categories of LT patients have been reported. Interestingly, tumoral indication showed very good results, substantially in line with other common LT indications like cirrhosis (log-rank *P* = 0.89) or cholestatic diseases (log-rank *P* = 0.83). In detail, 5-and 10-year survival rates for any tumoral indication were 65.9% and 54.2%, respectively.

**Fig. 4:**
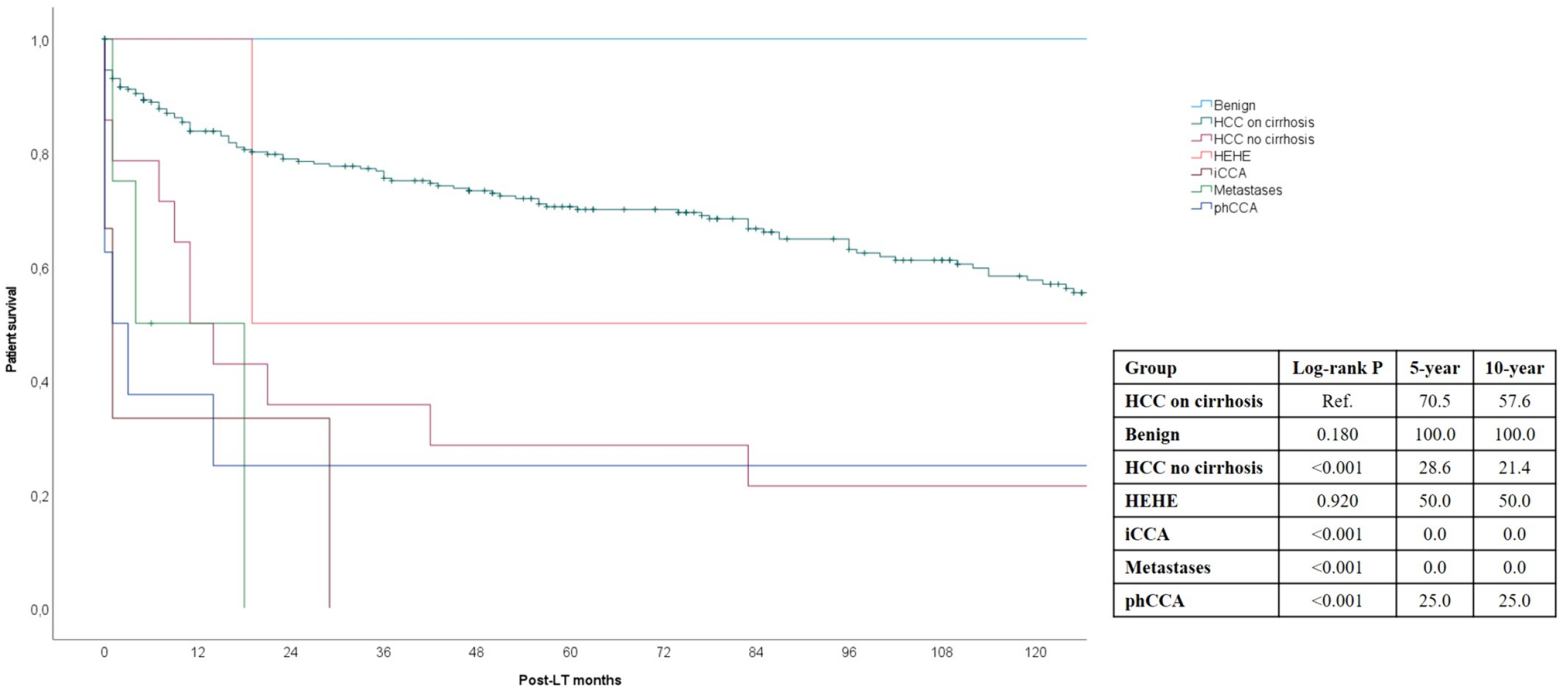
Long-term survival rates stratified according to the different tumor-related indications for LT

Stratifying the tumoral cases according to the different pathologies, the well-recognized indication of HCC on cirrhosis confirmed its excellent results, with 5- and 10-year survival rates of 70.5% and 57.6%, respectively. Only the benign indications and HEHE showed similar excellent results, with three cases of long survivors after transplant. All the other indications showed poor results. Patients with HCC on non-cirrhotic liver showed 5- and 10-year survival rates of 28.6% and 21.4%, respectively (log-rank *P* < 0.0001). Among the cases of phCCA, two long survivors were reported, with 5- and 10-year survival rates of 25.0% and 25.0%, respectively (log-rank *P* < 0.0001). No one case with iCCA or metastatic disease survived at least 5 years (Fig. 4).

## Discussion

This retrospective analysis reported contrasting results in transplanting patients with uncommon hepatic tumoral indications. Overall, the number of patients transplanted in the present series for oncological indications has increased from 8.8% in the 1980s to 47.1% in 2020–2023. However, the leading advancements in transplant oncology have primarily focused on patients with HCC on cirrhotic livers. Conversely, LT for uncommon tumors is still considered a relative contraindication based on the poor OS reported in the literature. It is only recently, with stringent patient selection and new protocols, that a modest increase in the number of patients transplanted for uncommon tumoral indications has been observed.

As for the HCC, the present series confirmed that most LTs were performed for HCC on cirrhosis, with excellent 5- and 10-year OS rates of 70.5% and 57.6%. These data align with the results reported in the literature [16]. Conversely, the series of 14 patients with HCC on non-cirrhotic liver showed poor results (i.e., 5- and 10-year OS rates of 28.6% and 21.4%). These harmful data are in line with the pieces of evidence from literature, in which the patients with HCC on non-cirrhotic liver typically present worse survival results. A large European series of 105 patients showed a 5-year OS rate of 49%, identifying macrovascular invasion, lymph node involvement, and the time interval between liver resection and transplantation < 12 months as independent risk factors for recurrence [4]. The tumor-free survival rate at 5 years was 43%, reporting similar results with respect to the 57.1% of recurrences observed in our series. A clear explanation for these unfair results is that almost the totality of the patients in the present series had a greater tumor burden with respect to HCC on cirrhosis patients; in detail, more than 70% of cases had a total sum of tumor dimensions exceeding 8 cm, and more than 40% had a macrovascular invasion.

Moreover, it is particularly relevant to note that the definition of “healthy liver” was also discussable. When we retrospectively explored the characteristics of the non-tumoral liver tissue, we noted that more than 50% of patients had mild-to-severe steatosis and inflammation rates. Grade 1–3 fibrosis was also reported in more than 40% of patients (Table 2). These data suggest that HCC on “normal livers” actually arose in patients with metabolic dysfunction-associated steatotic liver disease (MASLD) in a period in which this type of disease was not already encoded [17]. It is well known that the occurrence of MASLD-related HCC in patients without cirrhosis is increasingly recognized and poses a significant challenge regarding cancer surveillance [18]. Moreover, patients with HCC on MASLD typically present higher rates of tumor aggressiveness and recurrence [19]. All of these evidences justify the unpaired results observed in our series, suggesting a cautious approach to the transplant management of these patients.

As for the phCCA, our series reported eight cases performed in the 1990s. Historically, LT for phCCA yielded a 5-year OS of 25–30% [20]. The great revolution in this setting happened when the Mayo Clinic Protocol was developed in 1993, combining the benefits of radiotherapy, chemosensitization, liver transplantation, and appropriate patient selection for patients with localized, unresectable phCCA. Thanks to this approach, the Mayo Clinic group has reported 5-year OS rates exceeding 70% [21]. No patient received a neoadjuvant approach in our series but directly received LT. As expected, the results were poor, mainly considering that 25% of patients received a cluster/multivisceral transplantation. Despite the poor results observed (i.e., 5- and 10-year OS of 25%), it is relevant to note that 25% of the patients transplanted with this indication were long survivors, exceeding 12 years of post-LT follow-up. Only one recurrence was reported among the eight transplanted cases, with all the other graft losses caused by the premature death of the patients for non-tumoral causes. This evidence has been well clarified in a paper from the ELTR, in which the European experience of LT for CRLM performed during the 90 s was investigated, showing that 44% of graft loss or patient deaths were unrelated to tumor recurrence. The authors concluded that the dramatic post-LT progress in patient survival observed over the last 20 years should justify the prediction that survival rates for CRLM today would exceed the outcomes of past experiences [11]. Such a concept should be translated to the field of phCCA. In our small series, it was suggestive to note that the two long-surviving patients had a well-differentiated grading and no metastatic spread to the lymph nodes, further confirming the relevance of biological features of aggressiveness in the prediction of post-transplant survival [22].

As for iCCA, only three cases were reported in our series: two cases were transplanted during the 90ies, having as a leading indication the iCCA, while the last case was an iCCA transplanted with a preoperative erroneous diagnosis of HCC. The presence of an iCCA has been considered for a long time as an absolute contraindication for LT. Only recently, the studies from Sapisochin et al. showed promising results in patients (i) with very early iCCA (single tumor, ≤ 2 cm) on cirrhosis or (ii) with unresectable locally advanced iCCA [23]. In the international series reported, early iCCA showed 5-year survivals > 70%, suggesting that very well-selected cases can give reasonable results. Unfortunately, it was impossible in our series to evaluate the real risk of recurrence because 2/3 of cases died within 1 month from LT for non-tumoral reasons.

In the present study, three patients were transplanted for unresectable CRLM. Two were transplanted in the 1990s, both dying within the first 4 months following LT due to non-tumoral complications. Such a phenomenon has already been observed in the other tumors in the present and international series [11]. The already reported experience from the ELTR showed poor results in patients transplanted in the 80–90 s for uncommon transplant oncology indications, mainly due to the lack of advanced management and surgical evolutions [11]. The promising results of the new protocols for curing CRLM were first reported in the pivotal study published by the Oslo group in 2013: the SECA-I study reported 5-year survival rates of 60% [24].

Furthermore, the refined selection parameters reported in the SECA-II study consented to reach 5-year survival rates of 83% [25]. LT for CRLM remains limited to experimental protocols, but results from ongoing trials are expected to shift the paradigm significantly. Interestingly, the only patient of the present series transplanted during the last decade received a LT under the severe rules of the MELODIC protocol, showing excellent results at 18 months of follow-up [26].

According to the results of several recently published papers, a growing international interest has been observed in the non-conventional oncological indications to transplantation in the last 10 years [2, 22, 25]. Such a datum was not reported in our series, in which, as reported, only one case with an uncommon indication (i.e., CRLM) has been transplanted in recent years. A possible explanation for such evidence is that the selection criteria proposed for these uncommon indications are particularly severe, impacting the relatively small number of enrollable cases. Moreover, some new indications for LT have been proposed only during the last 10 years, and some time is required before they become routinely adopted worldwide. We are confident that, during the 2020s, the number of transplanted cases for uncommon tumoral conditions will grow in our center.

The present study presents some limitations. First, a small number of patients in our series have been transplanted for uncommon tumoral indications. Overall, in many cases, our reported experience represents a combination of small case series (i.e., three cases of iCCA and CRLM, respectively, and two cases of HEHE). Such a limitation is commonly reported in all mono-centric experiences, in which uncommon indications often present small numbers. Large multicenter experiences are needed to solve this issue, increase the data pool, and obtain more substantiated statistical results.

The heterogeneous nature of the investigated cases regarding tumor indication, period of LT, surgical and anesthesiologic management, immunosuppressive regimens, and post-operative oncological strategies represents another limit. All of these aspects should add result biases. As already reported, in many cases, the poor results observed should be caused more by the “old” management made in the 90 s than by an effective tumor aggressiveness. Modern strategies like the identification of new biological features for tumor selection [27], the use of perfusion machines for better management of the graft quality [28], and a more correct classification of the risk stratification made with artificial intelligence [29] should help in improving the quality of the results.

## Conclusions

A progressive increase in patients receiving LT for uncommon oncological diseases is expected in the following years. This evolution can be attributed to advancements in all aspects of transplant oncology, including creating new protocols and implementing organ availability. Historical series report poor survival outcomes despite more recent data showing promising results. Hence, the decision to transplant these patients should be under the risk and overall benefit of the patient while being cognizant of organ shortage and mortality rates. The results of the ongoing protocol studies are expected to confirm the validity of the unconventional tumor indications.

## Data Availability

The datasets generated during and/or analyzed during the current study are not publicly available but are available from the corresponding author on reasonable request.

## Abbreviations

CRLM: Colorectal liver metastases
HAI: Histological Activity Index
HCC: Hepatocellular carcinoma
HEHE: Hepatic hemangioendothelioma
i-CCA: Intrahepatic cholangiocarcinoma
LT: Liver transplantation
MASLD: Metabolic dysfunction-associated steatotic liver disease
NASH: Non-alcoholic steatohepatitis
OS: Overall survival
ph-CCA: Peri-hilar cholangiocarcinoma
Q1-Q3: First-third quartiles
STROBE: Strengthening the Reporting of Observational Studies in Epidemiology
